# Snai2‐mediated upregulation of *NADSYN1* promotes bladder cancer progression by interacting with PHB

**DOI:** 10.1002/ctm2.1555

**Published:** 2024-01-18

**Authors:** Li‐Juan Jiang, Song‐Bin Guo, Zhao‐Hui Zhou, Zhi‐Yong Li, Fang‐Jian Zhou, Chun‐Ping Yu, Mei Li, Wei‐Juan Huang, Zhuo‐Wei Liu, Xiao‐Peng Tian

**Affiliations:** ^1^ State Key Laboratory of Oncology in South China Guangdong Provincial Clinical Research Center for Cancer, Sun Yat‐sen University Cancer Center Guangzhou China; ^2^ Department of Urology Sun Yat‐sen University Cancer Center Guangzhou China; ^3^ Department of Medical Oncology Sun Yat‐sen University Cancer Center Guangzhou China; ^4^ Department of Pathology Sun Yat‐sen University Cancer Center Guangzhou China; ^5^ Department of Pharmacology College of Pharmacy Jinan University Guangzhou China; ^6^ Biotechnological Institute of Chinese Materia Medical Jinan University Guangzhou China

Dear Editor,

1

Bladder cancer is recognised as the 10th most prevalent cancer worldwide, with men having a lifetime risk of 1.1% and women .27%.^1^, ^2^ The epithelial‐to‐mesenchymal transition (EMT) process plays a crucial role in the progression from non‐muscle invasive bladder cancer (NMIBC) to muscle invasive bladder cancer (MIBC).^3^ However, the molecular mechanisms underpinning EMT in the advancement of bladder cancer remain largely unexplored.

We initially utilised high‐throughput RNAseq method to identify differential gene expressions between MIBC and NMIBC tissues. Upon intersecting three distinct gene sets (three groups of differential gene between MIBC and NMIBC), we observed upregulation of PHB in MIBC tissues (Figures 1A and S1). Given the reported role of PHB as a scaffold protein, we proceeded to perform RIP‐seq to investigate PHB‐associated RNA on both tumour and adjacent normal tissues, and we identified numerous PHB‐associated RNAs, most of which were protein‐coding RNAs (Figure S2A). GO and heatmap analysis revealed that the proteins encoded by the enriched genes were widely distributed in the cells and participated in a variety of cellular processes (Figures S2B and S2C). Analysis of RNAs enriched in tumour tissues identified LINC01410, MIR339 and NADSYN1 mRNA as the most significant binding RNA of PHB with high‐fold changes and low *P* values (Figure 1B). PHB showed greater RNA binding activity in tumour tissues than paired adjacent normal tissues, and a conserved binding motif was identified (Figure 1C). Further RIP‐seq followed by PCR revealed NADSYN1 mRNA as the only RNA that exhibited increased PHB binding in all three paired tumour tissues and adjacent normal tissues (Figure 1D). The peak distributions of tumour samples tended to be enriched in the middle of the gene body, whereas the PHB binding peaks tended to be enriched near the transcription start site in the proximal part of the gene (Figures S2D and S2E).

**FIGURE 1:**
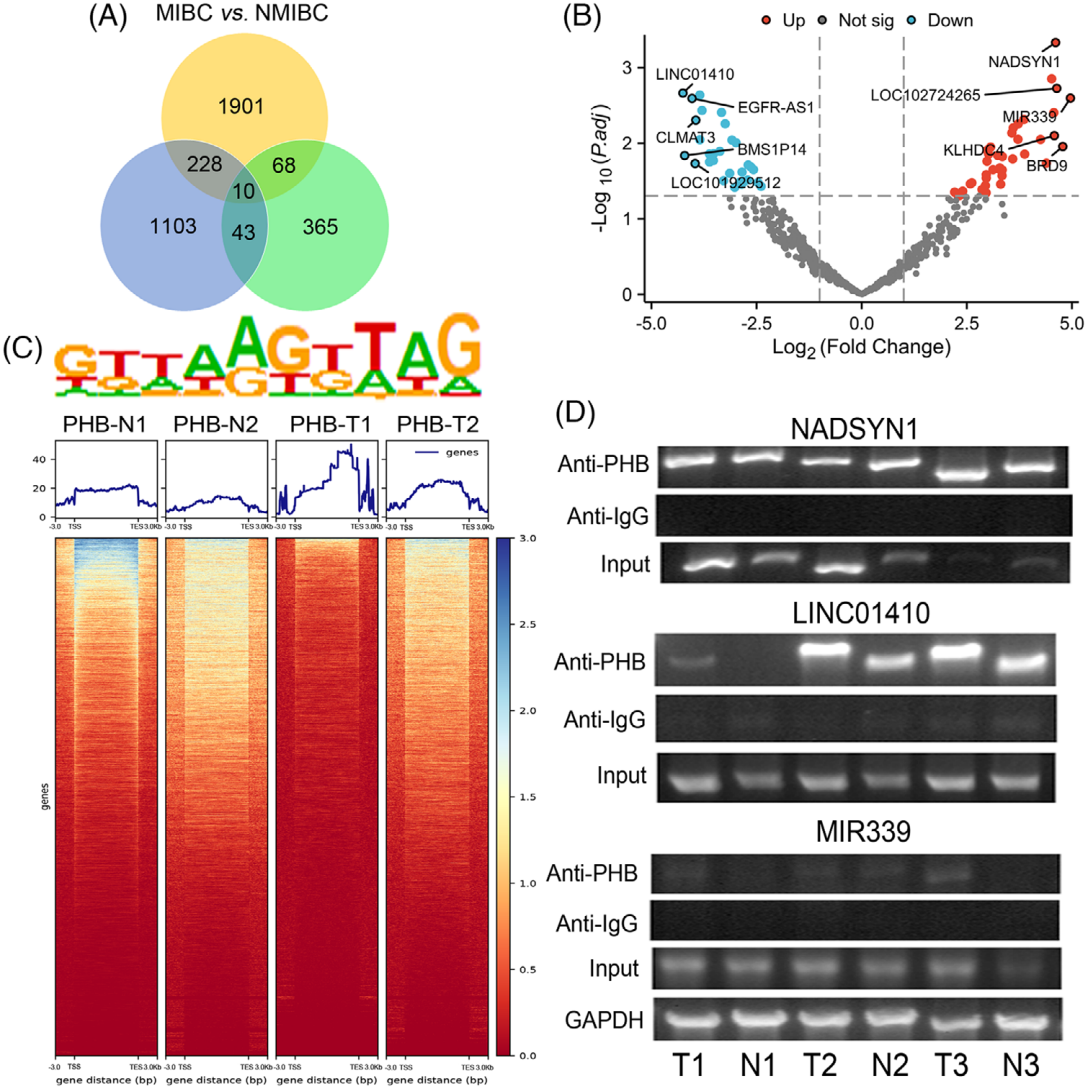
Delineation of RNA binding by PHB in paired bladder cancer tissues and adjacent normal tissues by RNA immunoprecipitation sequencing (RIP‐SEQ) assays. (A) The Venn diagram illustrates the variation in gene numbers between MIBC and NMIBC. By intersecting three different groups, we pinpointed 10 specific genes that exhibited differential expression. (B) Volcano map of PHB‐associated RNAs in tumour tissues versus normal tissues. (C) Motif analysis of enriched peaks in tumour sample. Peak distribution of genes in the gene body in tumour sample 1 (T1) and adjacent normal tissue sample 1 (N1), tumour sample 2 (T2) and adjacent normal tissue sample 2 (N2). (D) Representative agarose gel electrophoresis images of NADSYN1 (upper panel), LINC01410 (mid panel) and MIR339 (lower panel) in tumour and normal samples identified by RIP‐SEQ followed by PCR. MIBC, muscle‐invasive bladder cancer; NMIBC, non‐muscle‐invasive bladder cancer; FDR, false discovery rate. T, tumour tissue; N, adjacent normal tissue; PHB, Prohibitin. T3, tumour sample 3; N3, adjacent normal tissue sample 3.

PHB and NADSYN1 mRNA levels were positively correlated in bladder cancer tissues and cell lines (Figures S3A, S3B and S4). Immunohistochemical study demonstrated that both the protein levels of NADSYN1 and PHB were elevated in bladder tumour tissues, and they showed a positively correlated with each other (Figure S3C). Immunofluorescence analysis demonstrated that NADSYN1 mRNA was localised in both cytoplasm and nucleus (Figure S3D). Kaplan–Meier analysis indicated that higher NADSYN1 expression was correlated with a worse overall survival (OS) (Figure S3D).

We found that NADSYN1 expression positively correlated with bladder cancer progression‐related genes by using Gene Set Enrichment Analysis (GSEA) analysis of two independent datasets, GSE87304 and GSE128701 (Figure S5). PHB knockdown by specific siRNA led to a remarkable decreased in NADSYN1 expression, while PHB overexpression by lentiviruses upregulated NADSYN1 in bladder cancer cells (Figures S6A and S6B). PHB overexpression led to increase in the number of bladder cancer EJ and T24 cells, respectively, which was significantly abated by *NADSYN1* knockdown (Figures S6C and S6D). Furthermore, PHB overexpression induced a significant increase in the number of colonies, which was abolished by NADSYN1 knockdown (Figure S7A). Wound healing assays and trans‐well migration assays further demonstrated that PHB overexpression promoted the migration of bladder cancer cells, which was significantly lessened by NADSYN1 knockdown (Figures S7B and S7C). In nude mice, NADSYN1 knockdown significantly decreased the tumour size and reduced the number of metastatic foci sis ability, and tumour growth ability was inhibited (Figure S7D).

We constructed a series of PHB truncations (Figure 2A). RIP‐PCR revealed that PHB whose PHB domain was truncated failed to bind NADSYN1 mRNA (Figure 2B). We identified ammino acid residues 201−211 as an important region mediating PHB‐NADSYN1 interaction (Figure S8A).^4^ Compared to wildtype PHB or PHB with deletions in other domains, the deletion of the PHB domain led to a notable decrease in the expression of NADSYN1 (Figure S8B). Deletion of the PHB domain abrogated PHB‐induced clonogenic growth and migration of tumour cells (Figures 2A and B).

**FIGURE 2:**
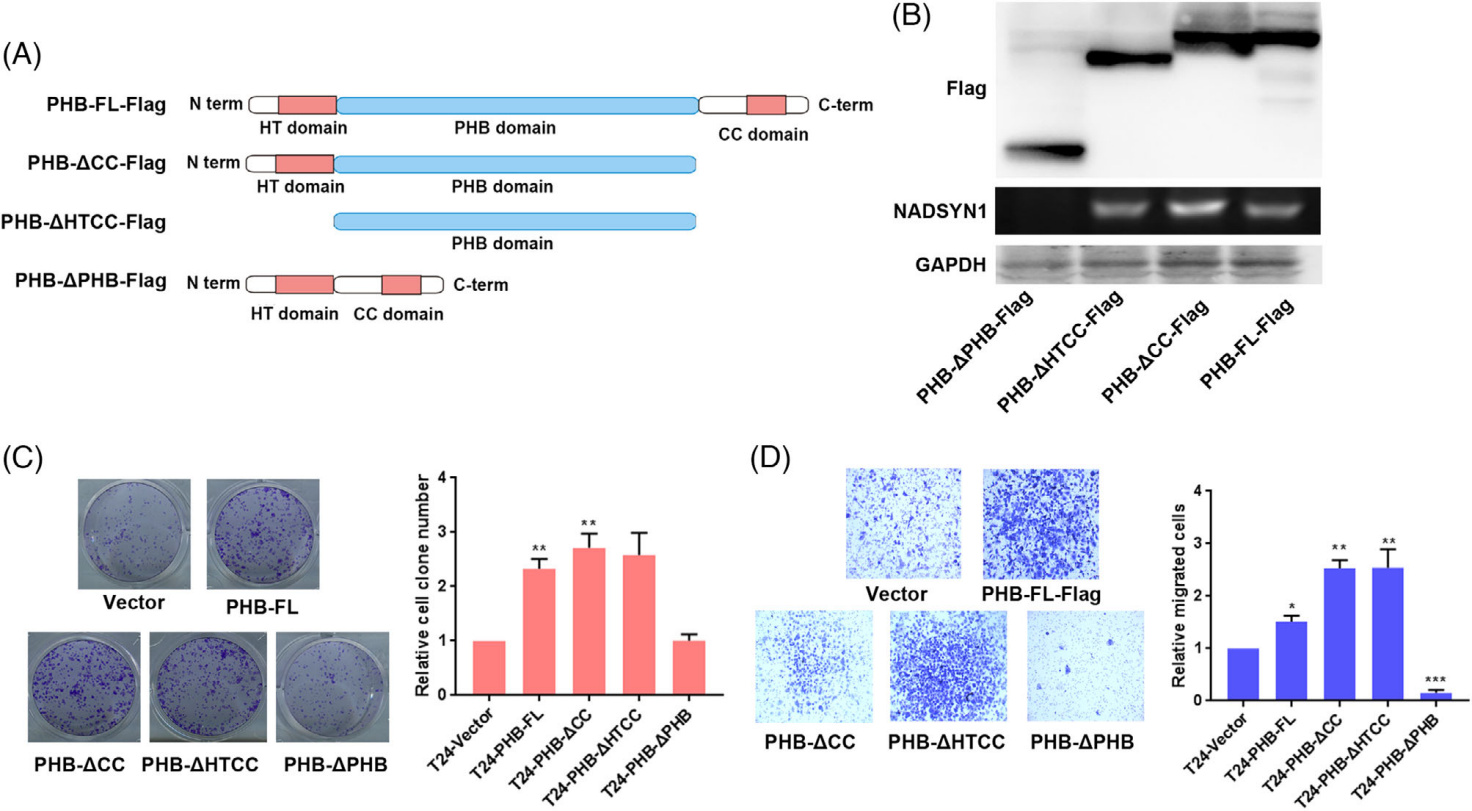
PHB domain is responsible for PHB‐NADSYN1 interaction. (A) Schematic diagram of PHB truncations. (B) Analysis of the binding of different isoforms of NADSYN1 by PHB. (C) and (D) Colony formation assay and trans‐well invasion assay were carried with cell lines. PHB‐△CC, PHB protein lacking CC domain. PHB‐△HTCC, PHB protein lacking HT domain and CC domain. PHB‐△PHB, PHB protein lacking PHB domain. * *P* < .05, ** *P* < .01, *** *P* < .001.

RNA‐Pull down assay demonstrated that mutation of the binding motif in NADSYN1 mRNA abolished PHB binding to NADSYN1 mRNA (Figure 3A). The addition of antisense nucleotide also abolished PHB binding to NADSYN1 mRNA (Figures 3A and S9A). PHB expression was nearly completely suppressed when NADSYN1 was knocked out in BJ cells, which demonstrated a feedback regulation of NADSYN1 on PHB (Figure S9B). PHB expression also declined in stable cells expressing NADSYN1 with mutated binding (Figure 3B). Mg132, a selective inhibitor of the ubiquitin mediated protein degradation system, reversed NADSYN1‐ko induced PHB downregulation (Figure 3B). However, PHB mRNA levels did not exhibit similar decreases when NADSYN1 was knocked out or mutated (Figure 3B). In *NADSYN1* knockout cells, PHB levels showed greater decrease with longer cycloheximide treatment, which, however, was not observed in WT cells (Figure S9C).

**FIGURE 3:**
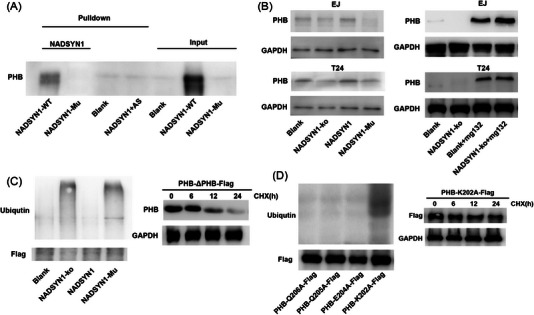
NADSYN1 promotes PHB stability in bladder cancer. (A) RNA pull‐down assay was carried with different combinations of RNAs indicated. (B) Western blotting assays were carried out in cell lines indicated. (C) Western blot analysis of ubiquitin in different cell lines indicated. The protein stability of PHB was valued in cells with different CHX treatment span. (D) Western blot analysis of ubiquitin in different cell lines expressing different mutants of PHB. PHB stability in cell lines expressing PHB K202A mutant.

Ubiquitin‐mediated protein degradation in *NADSYN1* knockout cells and NADSY N1 mutated cells was apparently increased (Figure 3C). More importantly, PHB‐ΔPHB degradation was increased (Figure 3C). Furthermore, K202A mutation led to significant PHB degradation (Figure 3D) and eliminated the effect of PHB on cell migration and clonogenic growth (Figure S9D).

Analysis using the PSCAN prediction tool revealed Snai2 and Snai3 binding to the promoter of *NADSYN1* (Figure S10A).^5^, ^6^ However, only Snai2 significantly elevated NADSYN1 expression, implicating Snai2 in NADSYN1 transcription (Figure S11A). Meanwhile, NADSYN1 positively correlated with Snai2 in both cell lines and tissues (Figure S11B). Our subsequent ChIP using anti‐Snai2 antibodies followed by PCR demonstrated binding of Snai2 to the *NADSYN1* gene region (Figure S10B). The luciferase assays further showed that Snai2 binding site mutation in the *NADSYN1* promoter abolished Snai2 activities (Figure S10B) and Snai2 knockdown attenuated NADSYN1‐mediated tumour growth and migration and PHB expression (Figures S11C and S11F). GSEA analysis showed a positive correlation between EMT and NADSYN1 in bladder cancer tissue samples (Figure S10C). TGF‐β increased TGFβR‐1/ALK5 inhibitor RepSox decreased NADSYN1 expression (Figure S11D).^7^ Immunohistochemical analysis of Snai2, NADSYN1, PHB, CDH1, and CDH2 demonstrated that in tissues with NADSYN1 high expression, the level of PHB, Snai2 and CDH2 was also high and the level of CDH1 was low (Figure S10D). Moreover, low Snai2 and NADSYN1 expression was associated with significantly longer survival of bladder cancer patients (Figure S11E).

In summary, our report describes EMT regulated the expression of NADSYN1 via Snai2 and Snai2‐NADSYN1‐PHB axis played a crucial role in bladder cancer progression (Figure S12).

## AUTHOR CONTRIBUTIONS

X‐PT design the study. L‐JJ, S‐BG, Z‐HZ, Z‐YL, F‐JZ, C‐PY, ML, W‐JH and Z‐WL obtained and assembled data. L‐JJ, S‐BG, Z‐HZ, and Z‐YL analysed and interpreted the data. X‐PT, Z‐WL and W‐JH wrote the manuscript. All authors reviewed the manuscript and approved the final version.

## FUNDING INFORMATION

This work was supported by grants from the National Natural Science Foundation of China (81802553, 81972382).

## CONFLICT OF INTEREST STATEMENT

The authors declare no competing financial interesting.

## ETHICS APPROVAL

Approval was obtained for all animal studies under the guidelines of Sun Yat‐sen University Cancer Center. All clinical study was approved by the Ethics Review Board of Sun Yat‐sen University Cancer Center (SYSUCC), and written informed consent was obtained from all subjects.

## ETHICS APPROVAL AND CONSENT TO PARTICIPAT

The approval of the current study was granted by the Institute Research Medical Ethics Committee of Sun Yat‐sen University Cancer Center. All the participants provided written informed consent and all of the cases were anonymised.

## Supporting information

Supporting Figures

## Data Availability

The RIP‐SEQ data have been deposited onto the NCBI Sequence Read Archive (SRA) database under the accession code SUB12394918. The RNAseq data have been uploaded into Gene Expression Omnibus (GEO) GSE243441.
