# Supplementary material for: Snai2‐mediated upregulation of NADSYN1 promotes bladder cancer progression by interacting with PHB
Source: Clin Transl Med. 2024 Jan 18;14(1):e1555. doi: 10.1002/ctm2.1555 (PMC10797243; doi:10.1002/ctm2.1555)
Supplement: Supplementary file 1 — Supporting Figures [file CTM2-14-e1555-s001.doc]

Supplementary Materials for

**Snai2-mediated upregulation of** ***NADSYN1* promotes bladder cancer progression by interacting with PHB**

Li-Juan Jiang^*^, Song-Bin Guo^*^, Zhao-Hui Zhou^*^, Zhi-Yong Li^*^, Fang-Jian Zhou, Chun-Ping Yu, Mei Li, Wei-Juan Huang^†^, Zhuo-Wei Liu^†^, Xiao-Peng Tian^†^

†Corresponding authors: Xiao-Peng Tian, M.D, PhD. Department of Medical Onology, State Key Laboratory of Oncology in South China, Provincial Clinical Research Center for Cancer，Sun Yat-sen University Cancer Center, No. 651, Dongfeng Road East, 510060 Guangzhou, China. Tel: 86-20-87342823. Email: [tianxp@sysucc.org.cn](mailto:tianxp@sysucc.org.cn).

**This file includes:**

Materials and Methods

Discussion

Abbreviation

Figure. S1 to S12

**Materials and methods**

**Bladder cancer cell lines and bladder cancer samples**

The bladder cancer cell lines, purchased from the American Type Culture Collection (Manassas, VA, USA), were cultured in suitable media. Bladder cancer tissues, along with fresh specimens of the same, were procured from the Cancer Center of Sun Yat-sen University. The collection of bladder cancer tissue samples was authorized by the Institutional Review Boards of Sun Yat-sen University Cancer Center, in compliance with the established guidelines for the use of human tissue specimens in research. All participants provided their written informed consent.

**Western blotting and immunoprecipitation assays**

Cell lysis was performed using an ice-cold RIPA buffer (Invitrogen, Carlsbad, CA, USA) with a mixture of protease inhibitors. The lysate underwent centrifugation at 13,000 rpm for 30 min at a temperature of 4°C. The protein concentration was ascertained using the BCA method. Following this, the proteins were subjected to SDS-polyacrylamide gel electrophoresis and then transferred onto polyvinylidene difluoride membrane (Pall, Port Washington, New York, USA). The membrane was later incubated with the primary antibody and subsequently with the secondary antibodies. The ECL detection system protein was employed to visualize the protein bands.

For immunoprecipitations, the cell samples were rinsed with PBS, then lysate without MG132 was introduced, and subsequently, the cell lysate was collected. The cell lysate was subjected to overnight incubation at 4℃ on a rotation wheel with anti-PHB antibody (normally Anti-Flag, Sigma). Following that, protein A/G magnetic beads (Bio-rad, 1614833) were introduced and incubated for a duration of 90 minutes, the immunoprecipitants in the eluate were examined by Western blotting assaying using the secondary antibodies.

**RNA interference and gene knockout**

In the context of RNA interference, cells were subjected to transfection with small interfering RNA (siRNA) that specifically targets NADSYN1 and Snai2, or alternatively with scrambled siRNA (Ambion, Austin, Texas, USA). This procedure was executed using the Lipofectamine 2000 reagent (Invitrogen), strictly adhering to the guidelines provided by the manufacturer. The sequences used were as follows: NADSYN1-siRNA-1, SS: GCAGAGUGAUAUUCCUCAACA, AS: UUGAGGAAUAUCACUCUGCAG; NADSYN1-siRNA-2, SS: GCUCCAGUGCGGACAUCAACC, AS: UUGAUGUCCGCACUGGAGCAG; NADSYN1-shRNA-1, SS: GCAGAGTGATATTCCTCAA; AS: TTGAGGAATATCACTCTGC; NADSYN1-shRNA-2, SS: CCTACAGCATGTTCTGCAA; AS: TTGCAGAACATGCTGTAGG; PHB-siRNA, SS: AGAUGUGAGUCCUGUUGAAGA, AS: UUCAACAGGACUCACAUCUCG, Snail2-siRNA-1, SS: GAGAUGUUGUCUAUAGCUAUG, AS: UAGCUAUAGACAACAUCUCAG; Snail2-siRNA-2, SS: GAAUGUCUCUCCUGCACAAAC, AS: UUGUGCAGGAGAGACAUUCUG; Snail3-siRNA, SS: CCUGCAAGUACUGCGACAAGG, AS: UUGUCGCAGUACUUGCAGGUG; PCR primer sequence of PHB, Forward: GTCAGAGTGGAAGCAGGTGAG, Reverse: GGACCCTCTCACACGCACG; PCR primer sequence of NADSYN1, Forward: GCAAAATGTGCAGGCTCGAA, Reverse: GCACTGGAGCAGTCGTACTT; PCR primer sequence of Snail2, Forward: CATCTTTGGGGCGAGTGAGT, Reverse: ATGGCATGGGGGTCTGAAAG; PCR primer sequence of Snail3, Forward, GCTTTGAGTGCTTCCACTGC, Reverse, AGTGCGGATGTGCATCTTGA.

For gene knockout, cells were transfected with lentiviruses pLentiCRISPRv2 harboring indicated gRNA sequences and selected with puromycin (Gibco, A1113803). Gene silencing and knockout were confirmed by Western blotting assays. Antisense NADSYN1 (NADSYN1+AS) and NADSYN1-mu were purchased from Tsingke Biotech. Note that NADSYN1-mu was synthesized with the interacting fragment (anti-PHB RIP_Peak of NADSYN1) of PHB and NADSYN1 deleted compared with original NADSYN1 mRNA.

**Wound healing assays**

In the wound healing assays, bladder cancer cells were arranged at a density of 100 cells per well in 12-well plates and were cultivated until they achieved 90% confluence. Wounds were initiated using a 100-μl pipette tip, and photographs were taken at predetermined time intervals.

**Colony formation assays**

As for colony formation assays, each well of a 12-well plate was populated with 800 cells. After an incubation period of 8 days, the cells were solidified with 70% ethyl alcohol and dyed with crystal violet. Plate images were obtained using the MFC-7340 scanner.

**Transwell migration assays**

In the case of transwell migration assays, bladder cancer cells were planted into the upper chamber of Matrigel invasion chambers. The lower chamber was enriched with FBS. Following a 48-hour period, the bladder cancer cells were solidified, dyed, and counted under a microscope. ^1^.

**Immunofluorescence staining**

Bladder cancer cells were cultivated on cover slides placed in 6-well plates until they achived a confluence of 40% to 60%. The specimens were solidified with 4% paraformaldehyde, and then underwent incubation with primary and secondary antibodies, subsequently followed by nuclear dyeing with DAPI (Invitrogen). Photographs were captured using an Olympus FV1000 system (Tokyo, Japan).

**Luciferase reporter assays**

The activity of the NADSYN1 promoter was evaluated using Luciferase reporter assays, as previously described ^2^. The cells were arranged into 96-well plates and transfected with the designated plasmids. The activities of FLuc and RLuc were evaluated using Dual-Luciferase Assay System (Promega, Madison, WI).

***In vivo* assays**

We acquired 20 male BALB/cnu/nu mice, each 4 weeks old, from Beijing Vital River Laboratory Animal Technology. These mice were accommodated in groups of four, having free access to both food and water. The mice were allocated into four groups, with each group being treated with the specified cell lines. siRNAs were administered every three days until the conclusion of the experiment. All procedures involving animals adhered to the guidelines set by the Committee on Animal Care at Sun Yat-sen University.

**Immunohistochemistry**

The tissue slides were deparaffinized with xylene, then rehydrated using gradient ethanol. This was succeeded by antigen retrieval using a citrate buffer with a pH level of 6.0, after which the slides were treated with 3% hydrogen peroxide. The slides were left to incubate overnight with antibodies against PHB, NADSYN1, or Snai2. Subsequently, the sliders were incubated with a secondary antibody using a universal two-step detection kit supplied by ZSGB-BIO (PV9000). The tissue slides were scanned using a 3D HISTECH Digital slice scanner. The intensity of the staining was evaluated on a scale from 0 to 3. Moreover, the proportion of cells that were positively stained was graded on a scale from 0 to 3 (0: 0-5%, 1: 6-25%, 2: 26-50%, and 3: >50%). These two scores were multiplied to calculate a composite staining score. A composite score ranging from 0 to 3 suggested low expression, whereas a score ranging from 4 to 9 suggested high expression.

**RNA sequencing**

Total RNA was extracted from fresh bladder tissues and used for library construction following the procedure outlined in the Illumina operating instruction (SMARTer Stranded Total RNA-Seq Kit v2). The concentration and distribution of cDNA library were assessed using Agilent 4200 bioanalyzer. Then the cDNA library sequence was detcted using Illumina NovaSeq6000 according to the manufacturer's instructions (Illumina). The raw reads were underwent filteration using Seqtk, then the raw reads were mapping with Hisat2 (version:2.0.4). Gene fragments were quantified using stringtie(v1.3.3b) and normalized using TMM (trimmed mean of M values). The edgeR software was employed to identify differentially expressed genes (DEGs), using a fold-change greater than 2 and a False Discovery Rate (FDR) value less than 0.05 as the criteria.

**RNA immunoprecipitation sequencing** (**RIP-SEQ) assays**

The RNA pull-down process was executed using a Pierce Magnetic RNA-Protein Pull-Down Kit, in accordance with the manufacturer's instructions. In brief, RNA was biotinylated using streptavidin magnetic beads. Following incubation with cell lysates, the proteins were eluted and analuzed by Western blotting assays. RIP-SEQ assays were performed using a Magna RNA-binding protein immunoprecipitation kit (Millipore, Bedford, MA). Human anti-PHB antibody (1:20, Abcam, ab181861) and isotype control IgG were used. Protein-RNA complexes were purified and immunoprecipitated RNAs were extracted with proteinase K before undergoing RNA-SEQ analysis.

**Statistical analysis**

Statistical evaluation was carried out using the SPSS statistical software (standard version 20.0). The Student's t-test was employed for the comparison of two groups. Survival curves were calculated using Kaplan-Meier analysis. A p-value less than 0.05 was considered statistically significant.

**Discussion**

EMT plays a vital role in cancer progression, characterized by the loss of cell adhesion and polarity. On a molecular level, cancer cells undergoing EMT are characterized by the upregulation of CDH2 (N-cadherin) and the downregulation of CDH1 (E-cadherin). Transcription factors responsible for EMT include Zeb1/2, Twist1/2, Snail2/3 and Slug^3^. In our prior research, we have shown that the overexpression of PHB in localized invasive bladder cancer independently predicts metastasis-free survival, suggesting that PHB may be crucial for EMT^4^. In the present study, we first uncovered a novel mechanism of Snai2-NADSYN1-PHB in BC progression, and indicated that PHB could serve as a therapeutic target for BC.

PHB belongs to a conserved protein family characterized by PHB domain, which is observed in several other scaffold proteins important for lipid raft associations, including flotillin, stomatin, and HflK/C. The coiled-coil domain at the C-terminal is typically associated with protein-protein interactions. ^5^. Our team, along with several others, have demonstrated the protein-protein interaction of PHB with other proteins. However, the role of PHB in RNA-protein interaction remains largely unexplored ^6^. Herein, we demonstrated for the first time that PHB is capable of RNA binding. Using two RNA-protein interaction prediction databases, we observed significant PHB-NADSYN1 mRNA interaction. Our subsequent RIP-SEQ, RIP-PCR and RNA-pull-down did demonstrate PHB-NADSYN1 mRNA interaction, which relies on the PHB domain of PHB as shown by RNA-protein interaction database analysis and RIP assays using truncated PHB. As PHB domain has been shown to play crucial roles in lipid raft function, this study fulfilled the function of PHB domain.

The level of PHB is higher in various types of cancer tissues compared to their adjacent normal tissues. However, the mechanism underlying this upregulation of PHB is largely unclear ^7^. In this study, we found a decrease in PHB protein degradation in bladder cancer tissues and cell lines. Ubiquitin-mediated protein degradation is responsible for physiological protein degradation and can be blocked by MG132 treatment ^8^. In this study, we observed decreased protein degradation in *NADSYN1* knockdown and knockout cells, indicating that PHB stability relies on NADSYN1. More importantly, the effect of NADSYN1 on PHB stability is tightly related to PHB-NADSYN1 interaction. Mutation of the binding motif of NADSYN1 abolished the effect of NADSYN1 on PHB stability.

NADSYN1 is an enzyme involved in the *de novo* synthesis of NAD, a molecule crucial for various biological processes including cell metabolism, proliferation, inflammation, and circadian rhythm. Abnormalities in NADSYN1 have been linked to birth defects affecting the vertebral, cardiac, renal systems and limbs ^9^. The exact role of NADSYN1 in cancer progression is still largely undefined. In our research, we demonstrated that NADSYN1 is overexpressed in bladder cancer tissues for the first time, and this upregulation is linked to enhanced tumor cell growth and migration. We speculated that the mechanism underlying the function of NADSYN1 may be related to tumor cell glucose metabolism and post modification of crucial proteins responsible for tumor aggressiveness via NAD. Furthermore, we revealed that Snai2, a key transcription factor of EMT, transcriptionally regulates NADSYN1. Snai2 positively influences the expression level of NADSYN1, thereby connecting the role of PHB to EMT. The limitation of this study is that there is no further development of new drugs targeting PHB protein for the treatment of bladder cancer. Anyway, the results provides a comprehensive explanation of our previous study on the correlation of PHB and tumor metastasis in bladder cancer samples.

Our report describes PHB could directly bind with NADSYN1 mRNA, and PHB domain to be responsible for PHB-NADSYN1 mRNA interaction. Moreover, degradation of PHB protein and NADSYN1 mRNA is inhibited by the interaction of PHB-NADSYN1. Finally, EMT regulated the expression of NADSYN1 *via* Snai2 and Snai2-NADSYN1-PHB axis played an important role in bladder cancer progression.

**Abbreviation**

PHB, Prohibitin; BC, bladder cancer; NAD, Nicotinamide adenine dinucleotide; NADSYN1, NAD synthetase 1; NMIBC, non-muscle-invasive bladder cancer; MIBC, muscle-invasive bladder cancer; EMT, Epithelial-to-mesenchymal transition; RIPA, Radio-immunoprecipitation assay buffer; BCA, Bicinchoninic acid; ECL, **Enhanced chemiluminescence; DAPI,** 4,6-diamino-2-phenyl indole; cDNA, complementary DNA; TMM, Trimmed mean of M values; DEGs, Differential expressed genes; FDR, False discovery rate; RIP-SEQ, **RNA immunoprecipitation sequencing;**  GO, **Gene ontology;** GSEA, **Gene set enrichment analysis;** TGF-β, transforming growth factor-β; TGFβR-1, **Transforming growth factor beta receptor I;** ALK5 inhibitor, TGF-Β RI kinase inhibitor II; CDH1, E-cadherin; CDH2, N-cadherin; Zeb, Zinc finger E-box-binding homeobox.


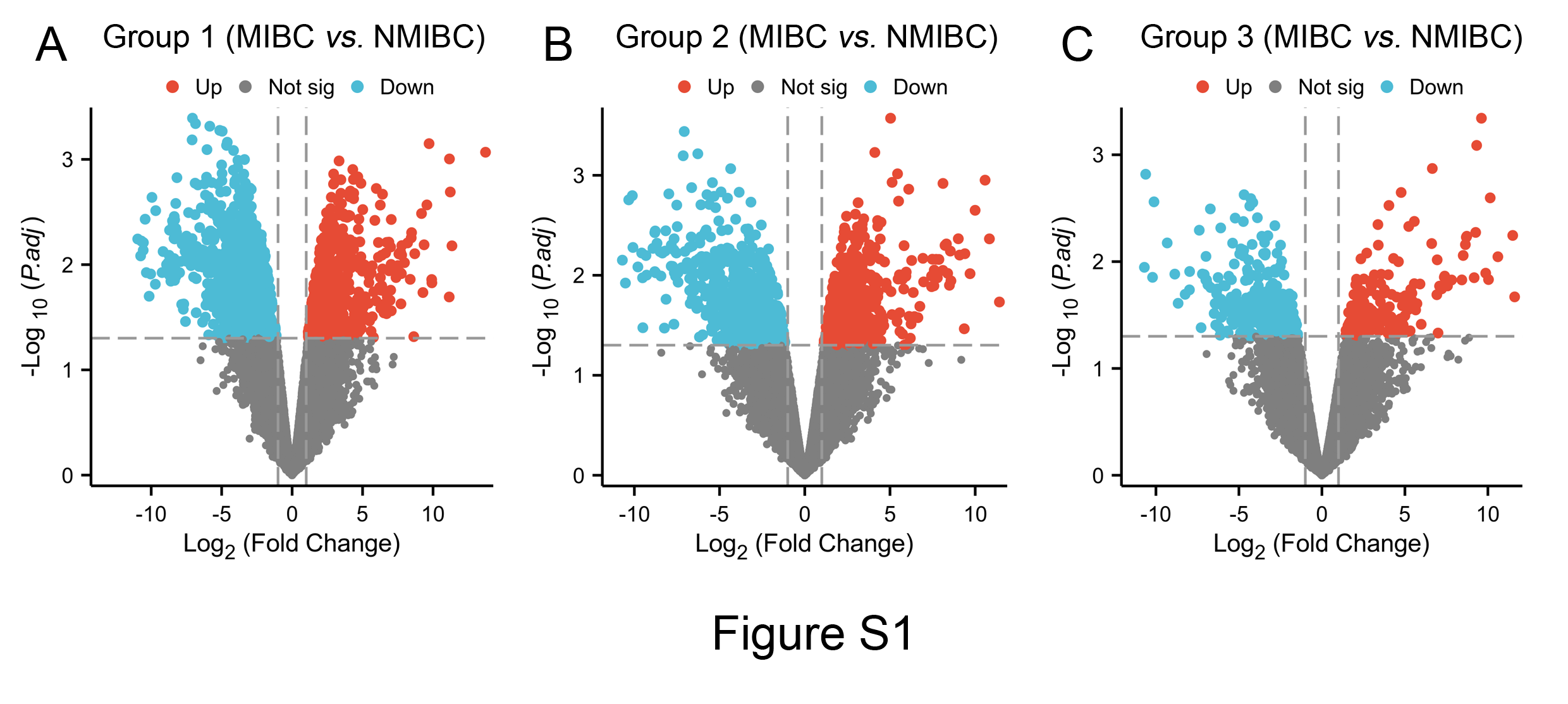


**Figure S1. Volcano map of different genes between MIBC tissues *versus* NMIBC tissues in three groups.**


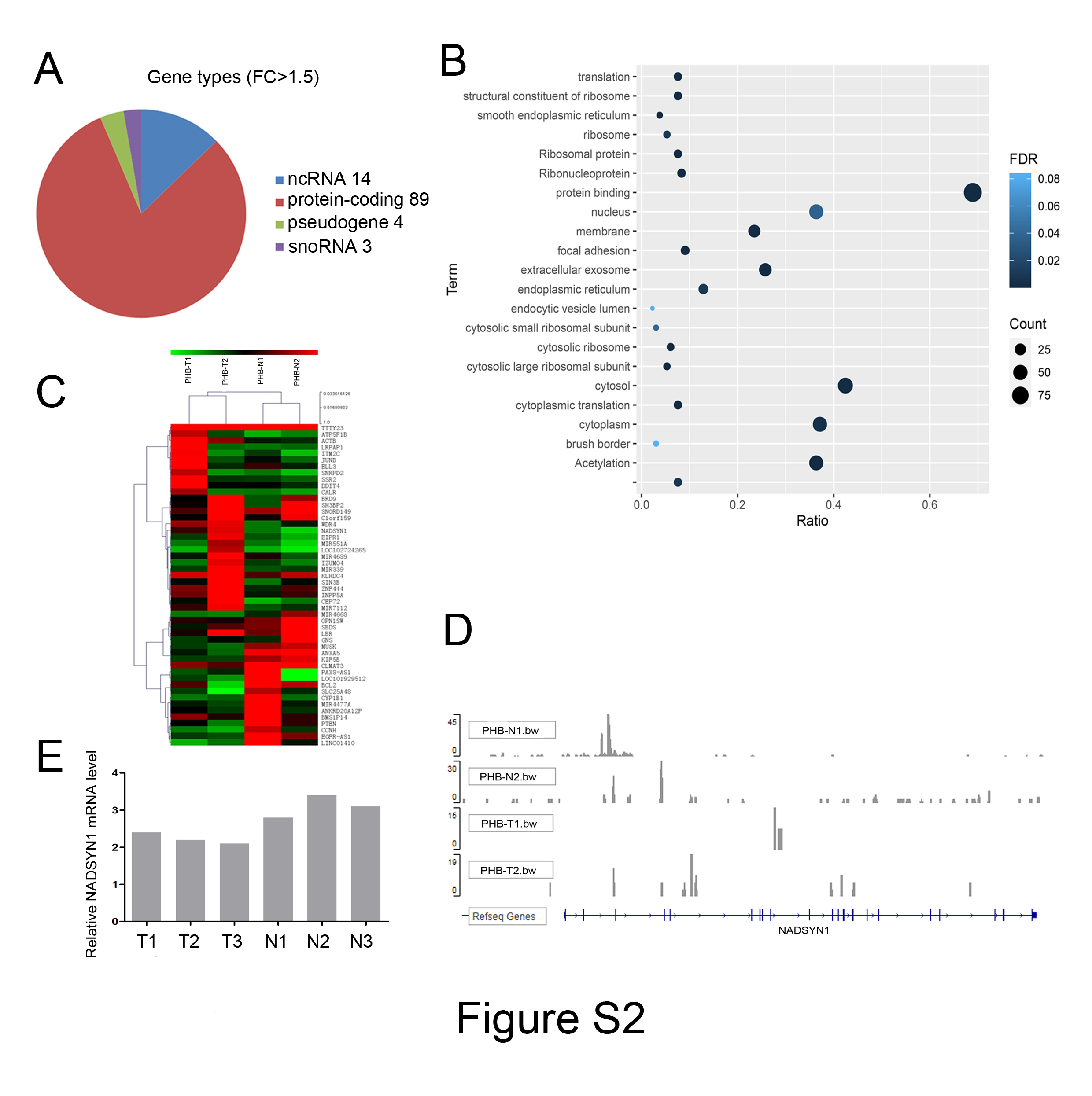


**Figure S2. NADSYN1 mRNA as a specific binding RNA of PHB**. Pie chart **(A)**, Gene Ontology analysis **(B)**, and heat map **(C)** of genes with >1.5-fold change in tumor tissues *versus* normal tissues. **(D)** Peak distribution of PHB binding peaks in tumor and adjacent normal tissues. FC, fold change. FDR, false discovery rate. T, tumor tissue; N, adjacent normal tissue. PHB, Prohibitin. **(E)** The relative of NADSYN1 mRNA bind to PHB.


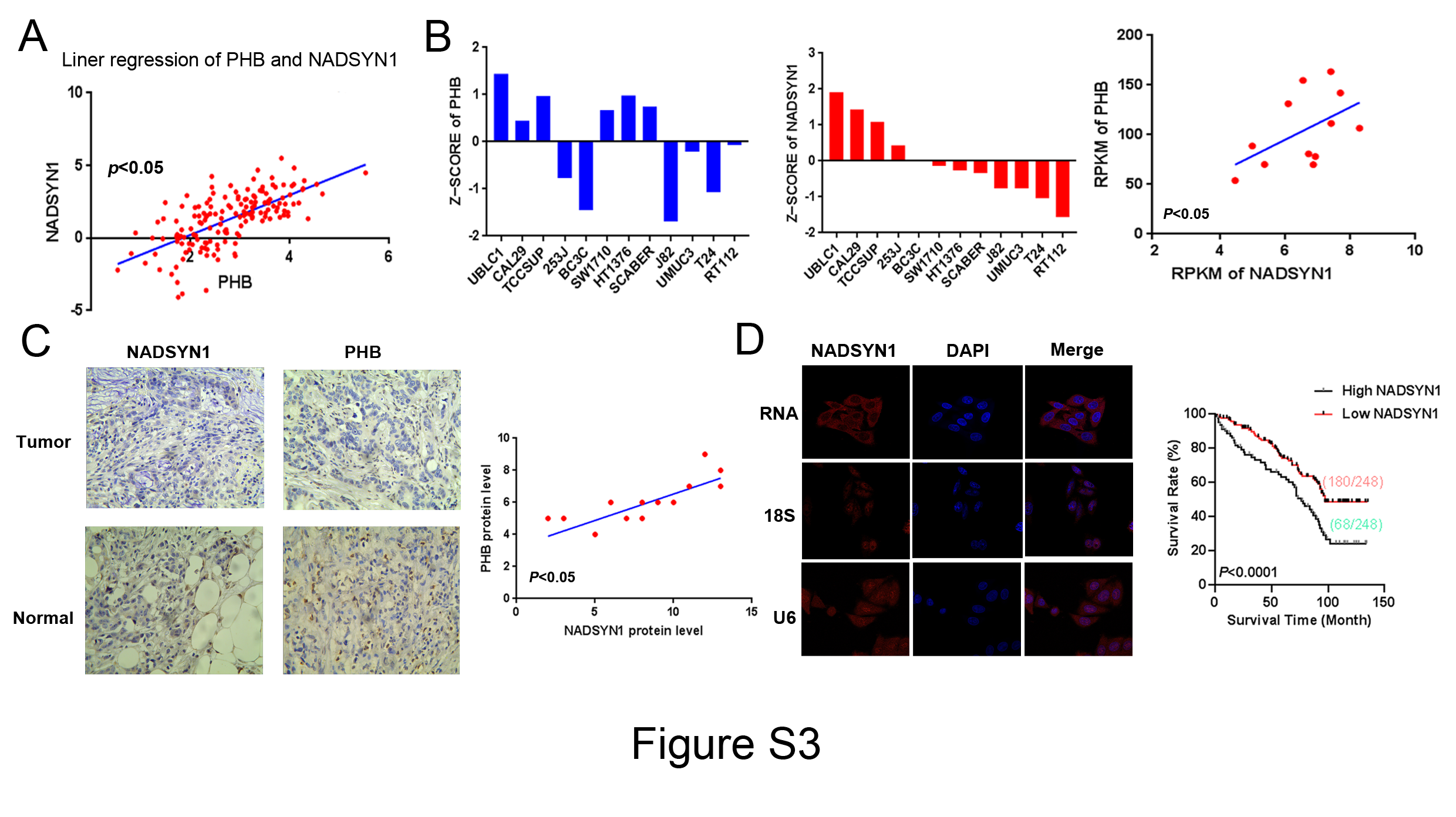


**Figure S3. NADSYN1 and PHB are overexpressed in bladder cancer tissues and associated with patient survival.** **A.** Linear regression of PHB and NADSYN1 mRNA levels of bladder cancer tissues in the Oncomine database. **B.** Expression of PHB and NADSYN1 mRNA in bladder cancer cells in Xena dataset. Linear regression analysis of correlation between PHB and NADSYN1 mRNA in cancer cells. **C.** Expression of PHB and NADSYN1 mRNA in bladder cancer tissues and normal tissues (n=15). Linear regression analysis of correlation between PHB and NADSYN1 mRNA in bladder cancer tissues. **D.** Immunofluorescence analysis of NADSYN1 mRNA in bladder cancer cell lines. Kaplan-Meier curve of OS of bladder cancer patients stratified by NADSYN1 expression.


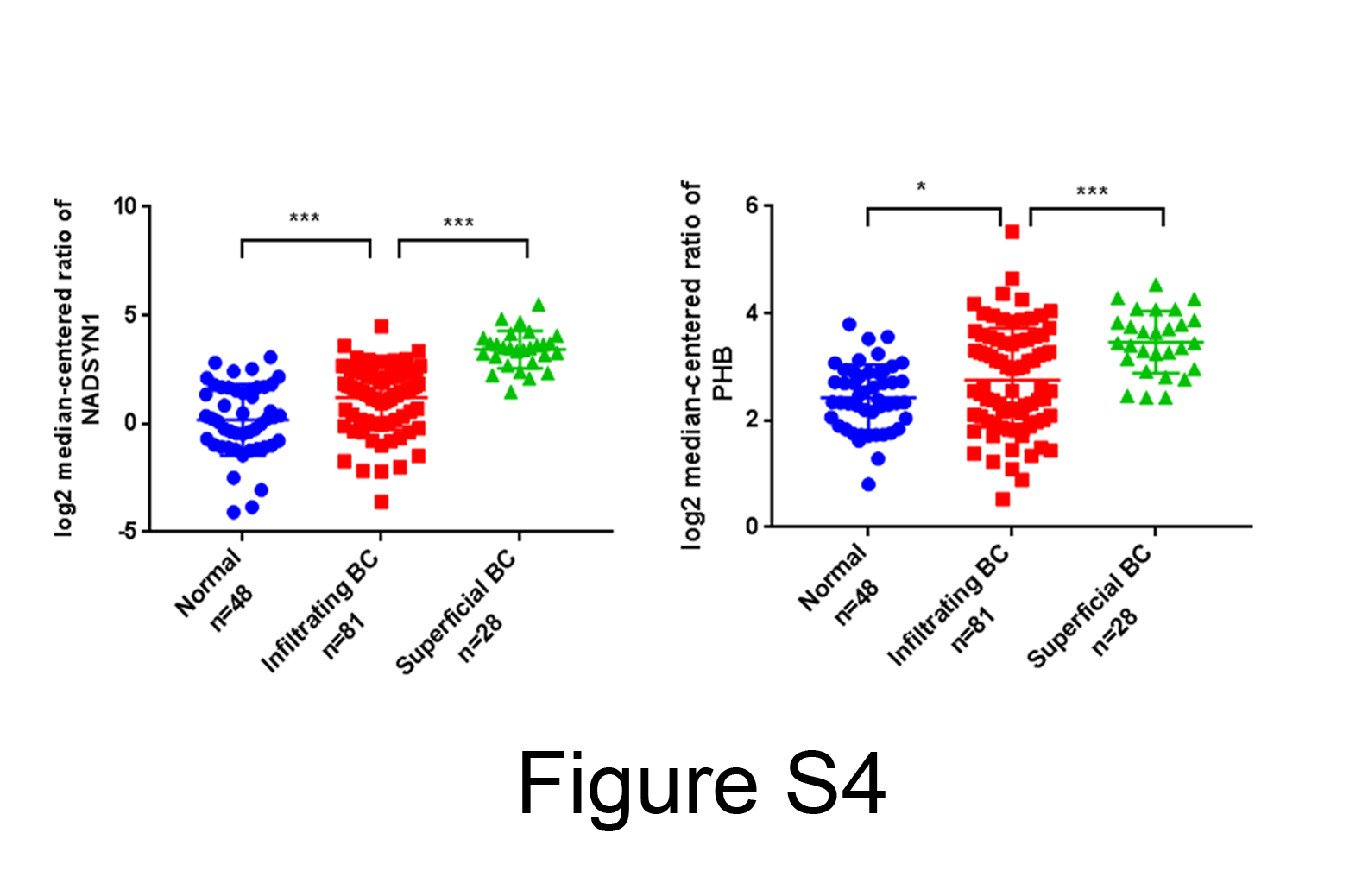


**Figure S4. Expression of NADSYN1 (left panel) and PHB mRNA (right panel) in bladder cancer tissues and normal tissues in the Oncomine database.** RPKM, **reads per kilobase of transcript per million reads mapped. BC, bladder cancer. *,** *P*<0.05. ***, *P*<0.001.


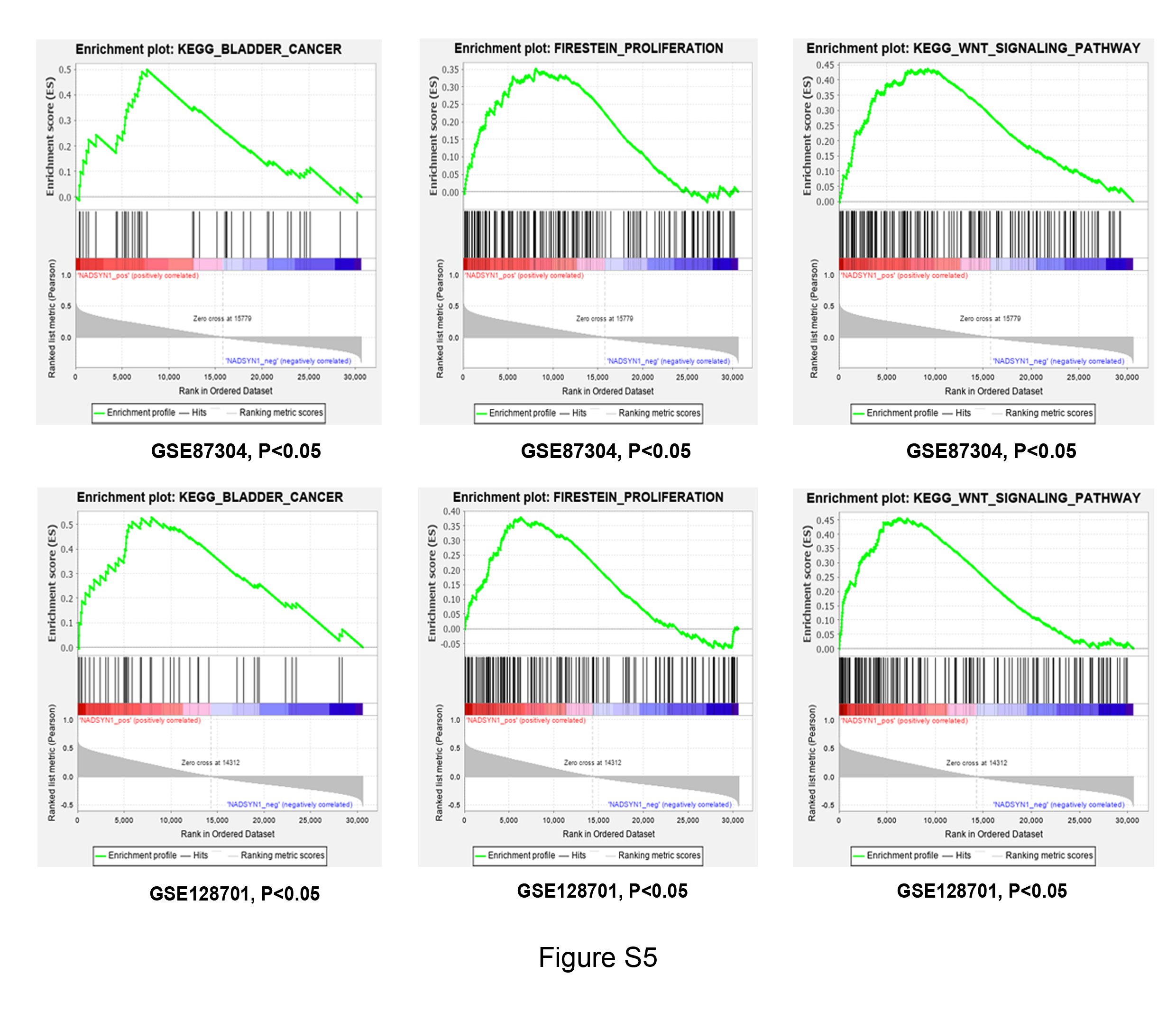


**Figure S5. Gene set enrichment analysis (GSEA) of NADSYN1 expression level in GSE87304 and GSE128701 with gene sets related to bladder cancer, proliferation and WNT signaling.**


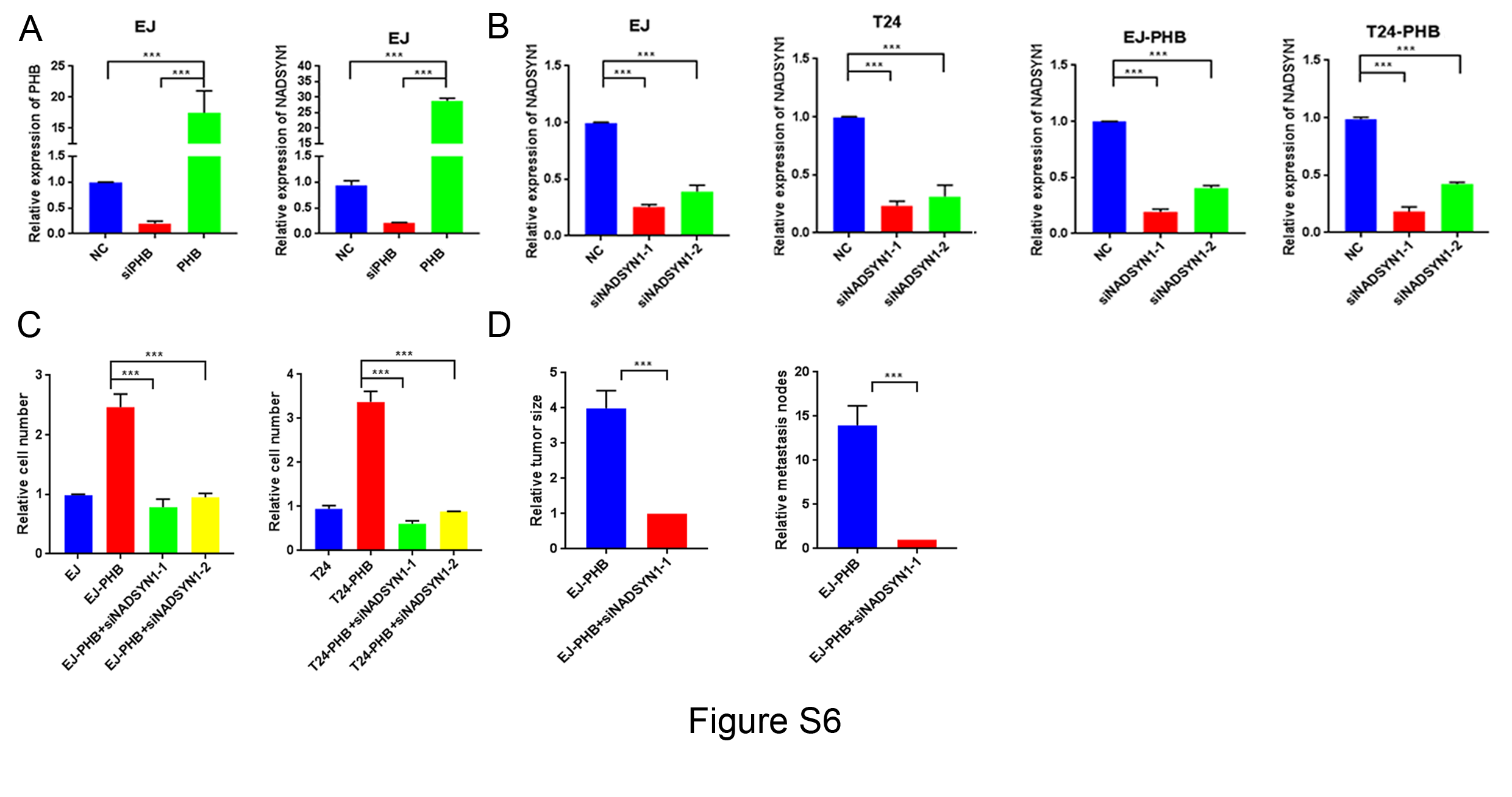


**Figure S6. The relative expression level of PHB and** **NADSYN1. A.** The expression level of PHB and NADSYN1 in siPHB bladder cancer cells. **B.** The expression level of NADSYN1 in siNADSYN1 EJ, EJ-PHB, T24, T24-PHB cells. **C.** The number of cells was counted with 72 h span in indicated bladder cancer cells. **D.** the relative tumor size and metastasis nodes in indicated bladder cancer cells. EJ-PHB, EJ cells that overexpress PHB, T24-PHB, T24 cells that overexpress PHB. siPHB, bladder cancer cells transfected with siRNA target PHB. siNADSYN1, bladder cancer cells transfected with siRNA target NADSYN1. ***, *P*<0.001.


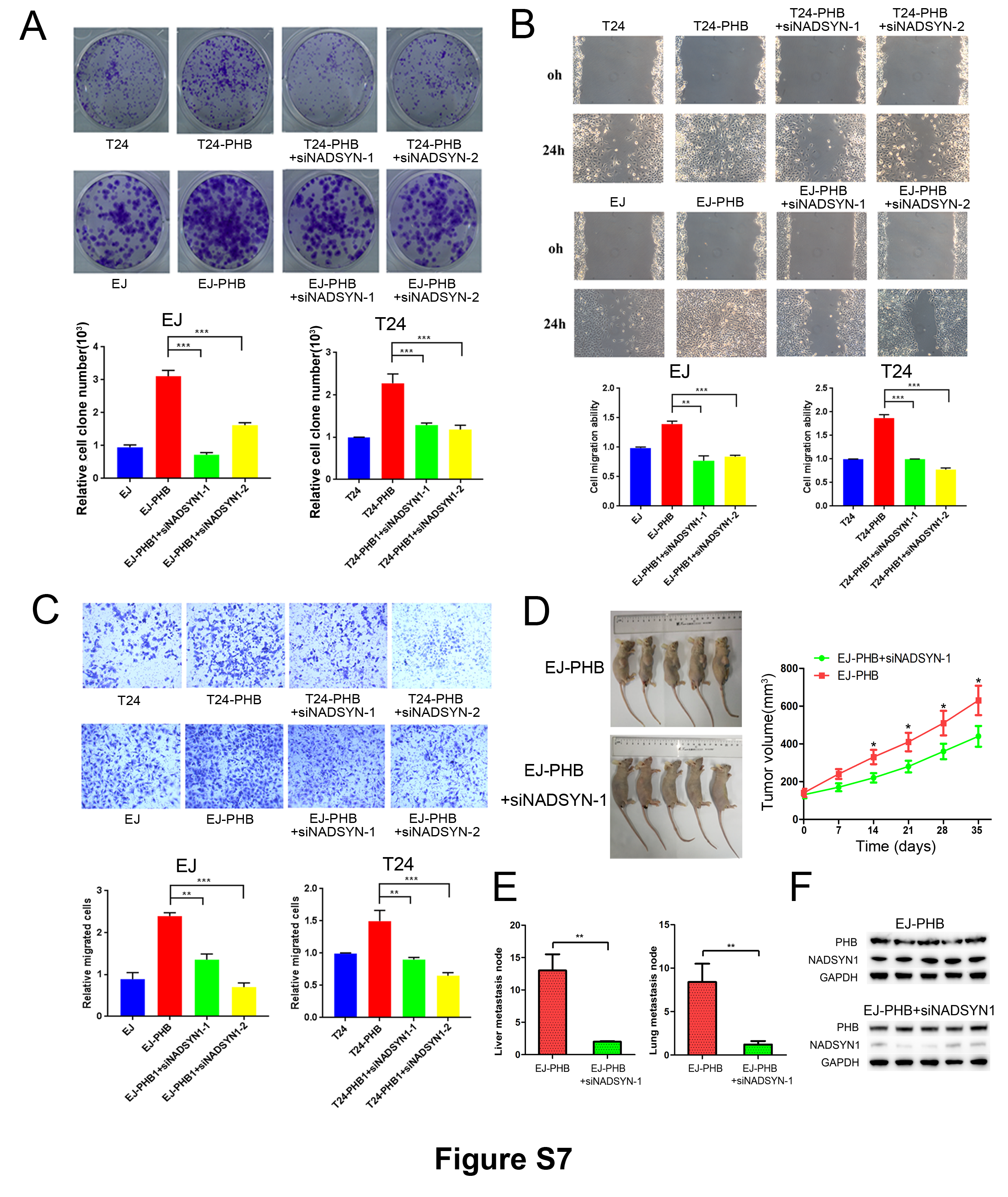


**Figure S7. NADSYN1 is indispensable for PHB-mediated tumor progression.** **A.** Colony formation assays were performed as described in methods and the number of colonies was counted. **B.** Wound healing assay was carried out as described in methods and wound span was measured at the indicated time points. **C.** Invasion assay was carried out as described in methods and migrated cells were stained and enumerated. **D.** Tumor volume (right) and growth curve (left) of xenograft tumor model. **E.**Nude mice were inoculated with the indicated cell lines *via* tail injection and liver and lung metastasis were evaluated. **F.** Expression status of PHB and NADSYN1 in xenograft tumor samples. Subcutaneous tumor formation was carried out with cell lines indicated. *, *P*<0.05; **, *P*<0.01; ***, *P*<0.001.


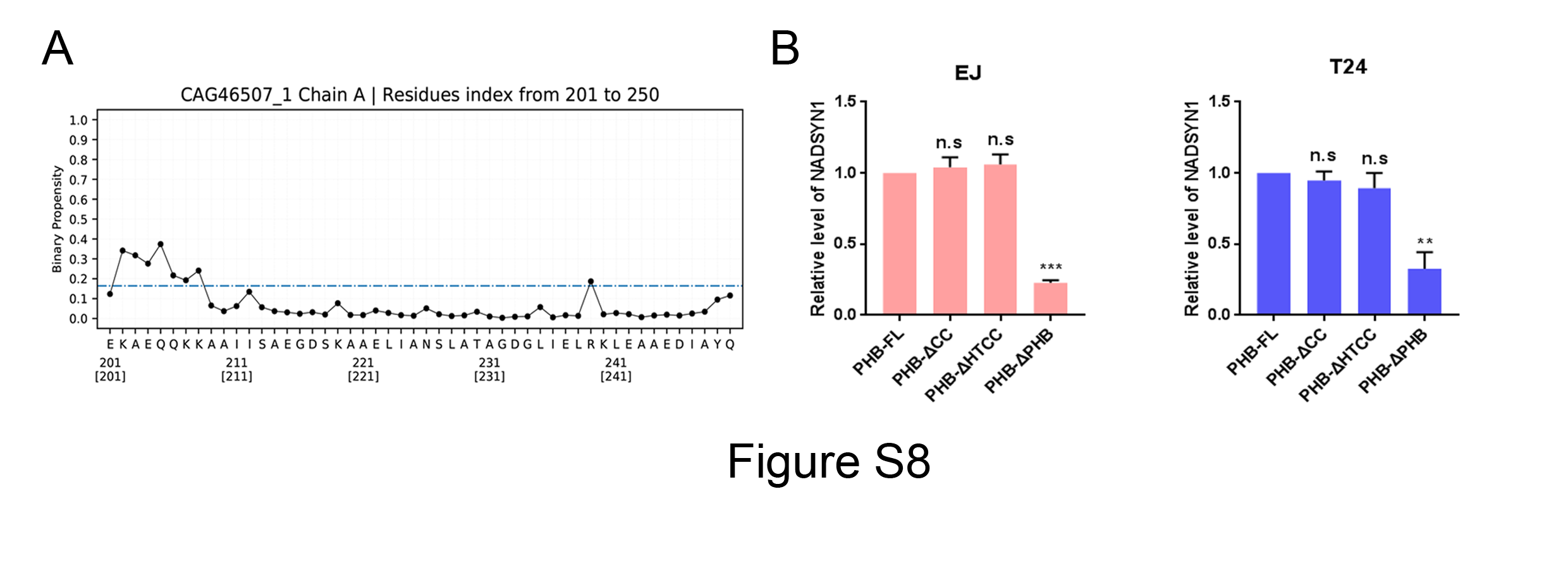


**Figure S8. The binding site of PHB binding to NADSYN1.** **A.** Prediction of amino acid residues participating in PHB binding to NADSYN1 mRNA using the aaRNA database. **B.** NADSYN1 mRNA expression was quantified in cell lines indicated. PHB-FL, PHB protein with flag label. PHB-△CC, PHB protein lacking CC domain. PHB-△HTCC, PHB protein lacking HT domain and CC domain. PHB-△PHB, PHB protein lacking PHB domain. n.s., no significant. ***, *P*<0.001.


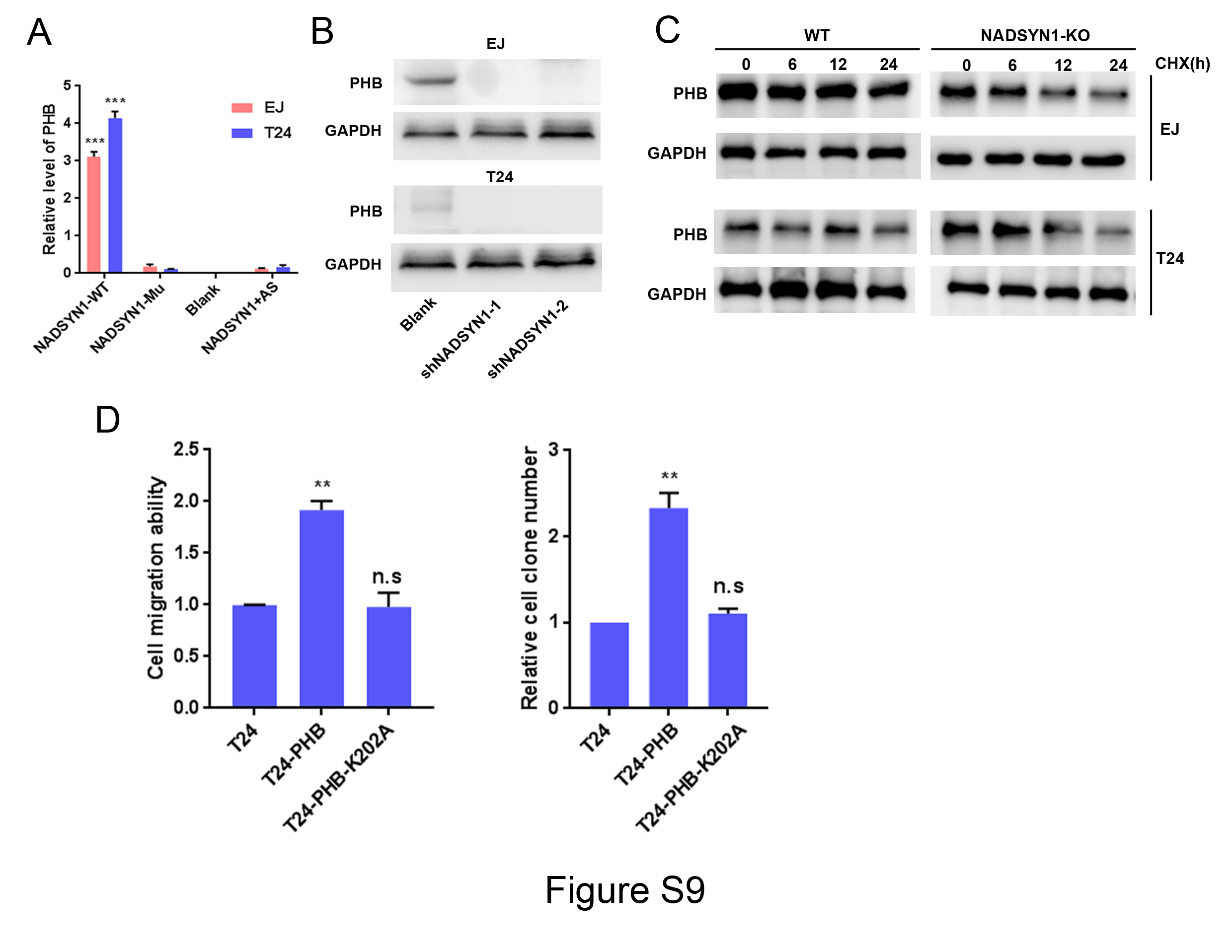


**Figure S9. The PHB protein can be stabilized by NADSYN1 mRNA. A.** The relative level of PHB in bladder cancer cells. **B.** Western blot analysis of PHB was carried out in cell lines indicated. **C.** The protein stability of PHB was valued in cells with different CHX treatment span. The cells were treated with cycloheximide for 6, 12 and 24 h. **D.** The relative cell migration ability and cell clone number of T2, T24-PHB, and T24-PHB-K202A cells. T24-PHB, T24 cells that overexpress PHB protein. T24-PHB-K202A, T24 cells that overexpress K202A mutant PHB protein. shNADSYN1, bladder cancer cells transfected with shRNA target NADSYN1. **, *P*<0.01; ***, *P*<0.001. n.s., no significant.


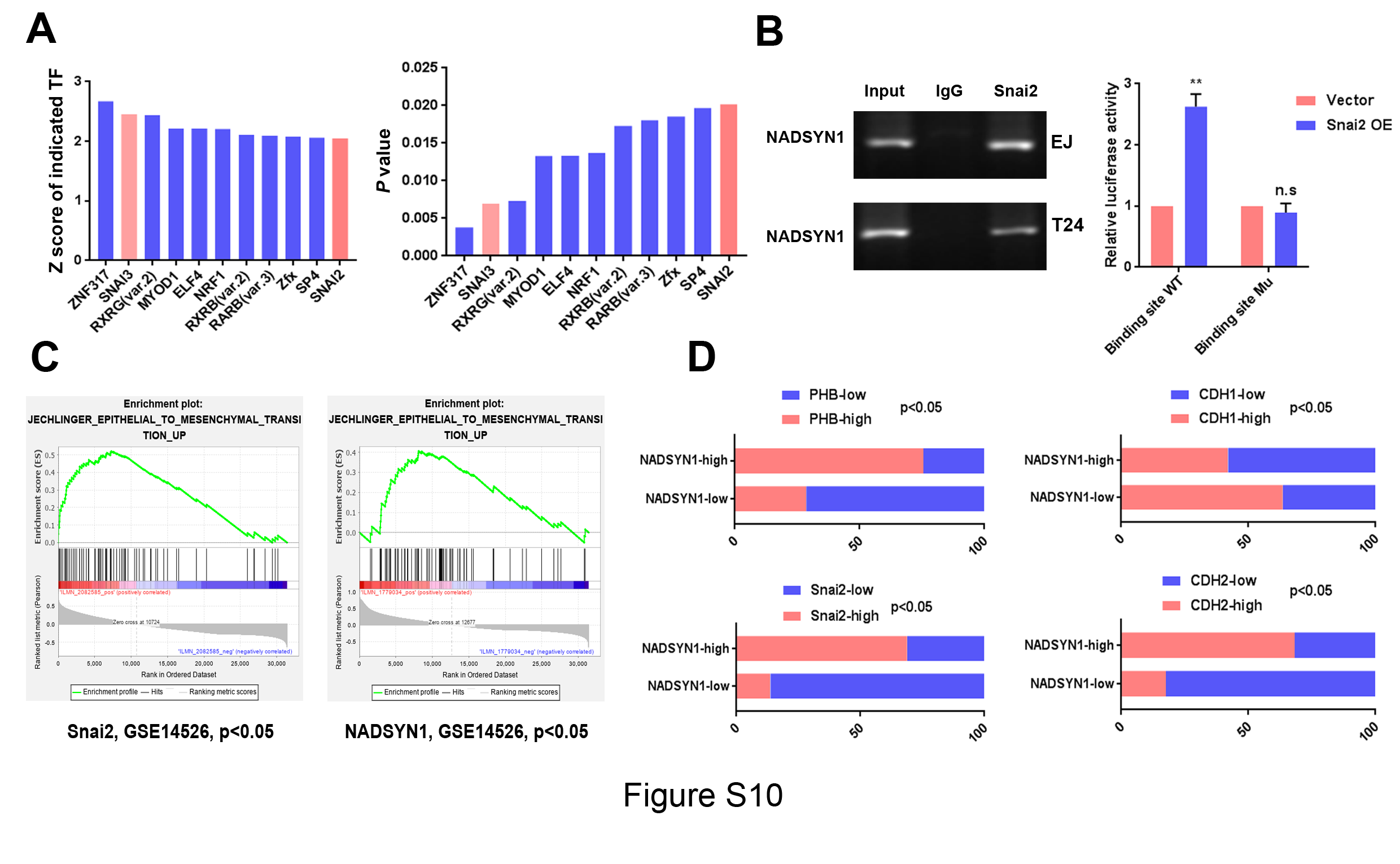


**Figure S10. NADSYN1 is transcriptionally regulated by Snai2**. **A** Pscan prediction of transcriptional factors in the promoter of NADSYN1. **B.** ChIP-PCR was carried out in NADSYN1 with the cell lines indicated. Relative luciferase activity of the promoter indicated. **C.** GSEA analysis of EMT gene set on Snai2 and NADSYN1 expression. **D.** The expression of PHB-NADSYN1-Snai2-CDH1/2 is shown. **, *P*<0.01. n.s., no significant.


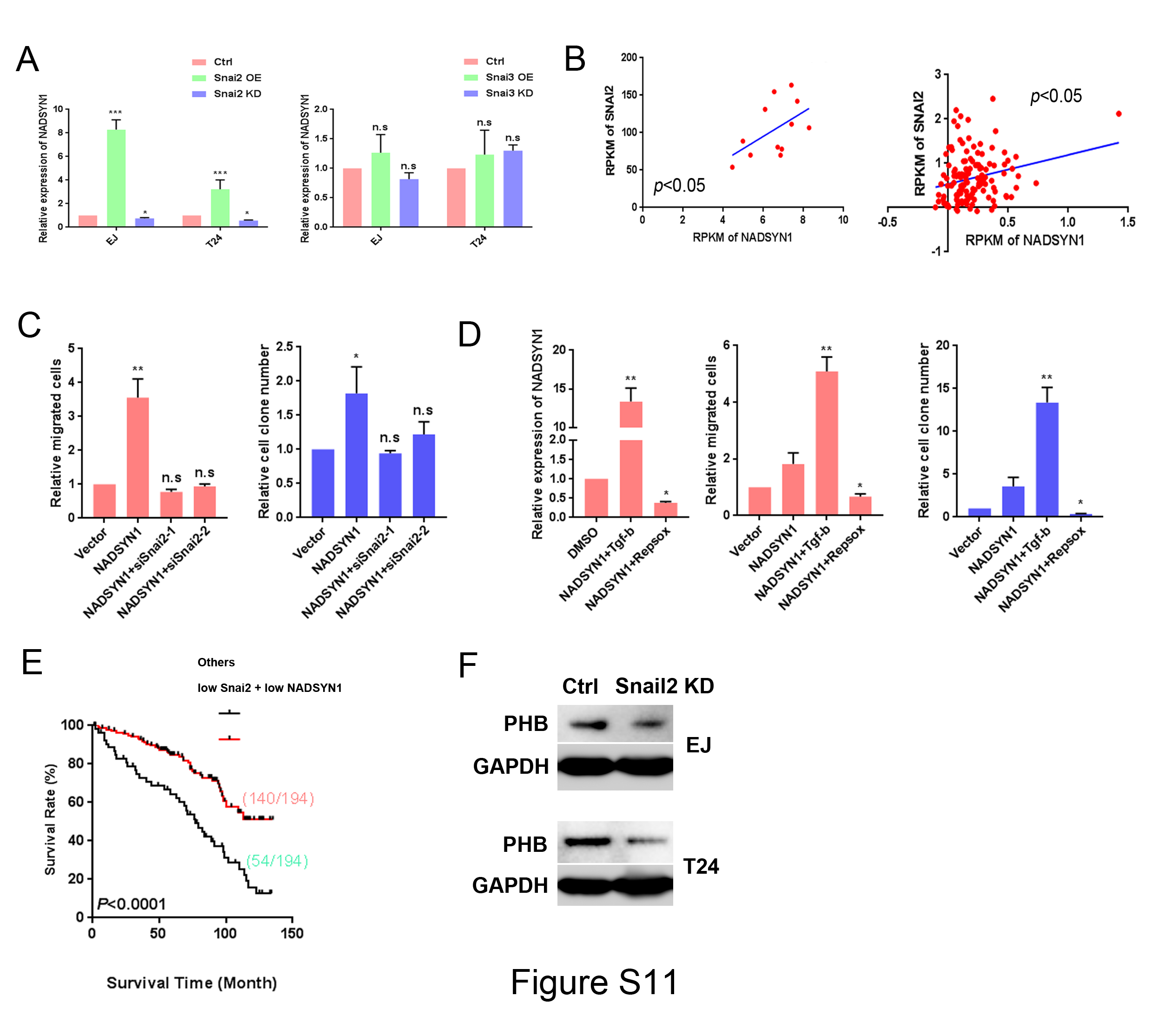


**Figure S11. NADSYN1 is a transcriptional target of Snai2. A.** The function of Snai2 and Snail3 on NADSYN1 expression. **B.** Linear analysis of Snai2 and NADSYN1 in bladder cancer cell lines and tissues samples (p<0.05). **C.** Cell migration and cell growth ability were evaluated in the cell lines indicated. **D.** The expression level of NADSYN1, cell migration and cell growth ability were measured in different bladder cancer cells treatment. **E.** Kaplan-Meier curves of survival of bladder cancer patients stratified by Snai2-NADSYN1 expression levels (high *vs.* low, *P*<0.001). **F.** The expression level of PHB was downregulated in Snai2 KD BJ cell lines. OE, overexpression. KD, knockdown. n.s., no significant. ***,** *P*<0.05. **, *P*<0.01; ***, *P*<0.001. n.s., no significant.

**
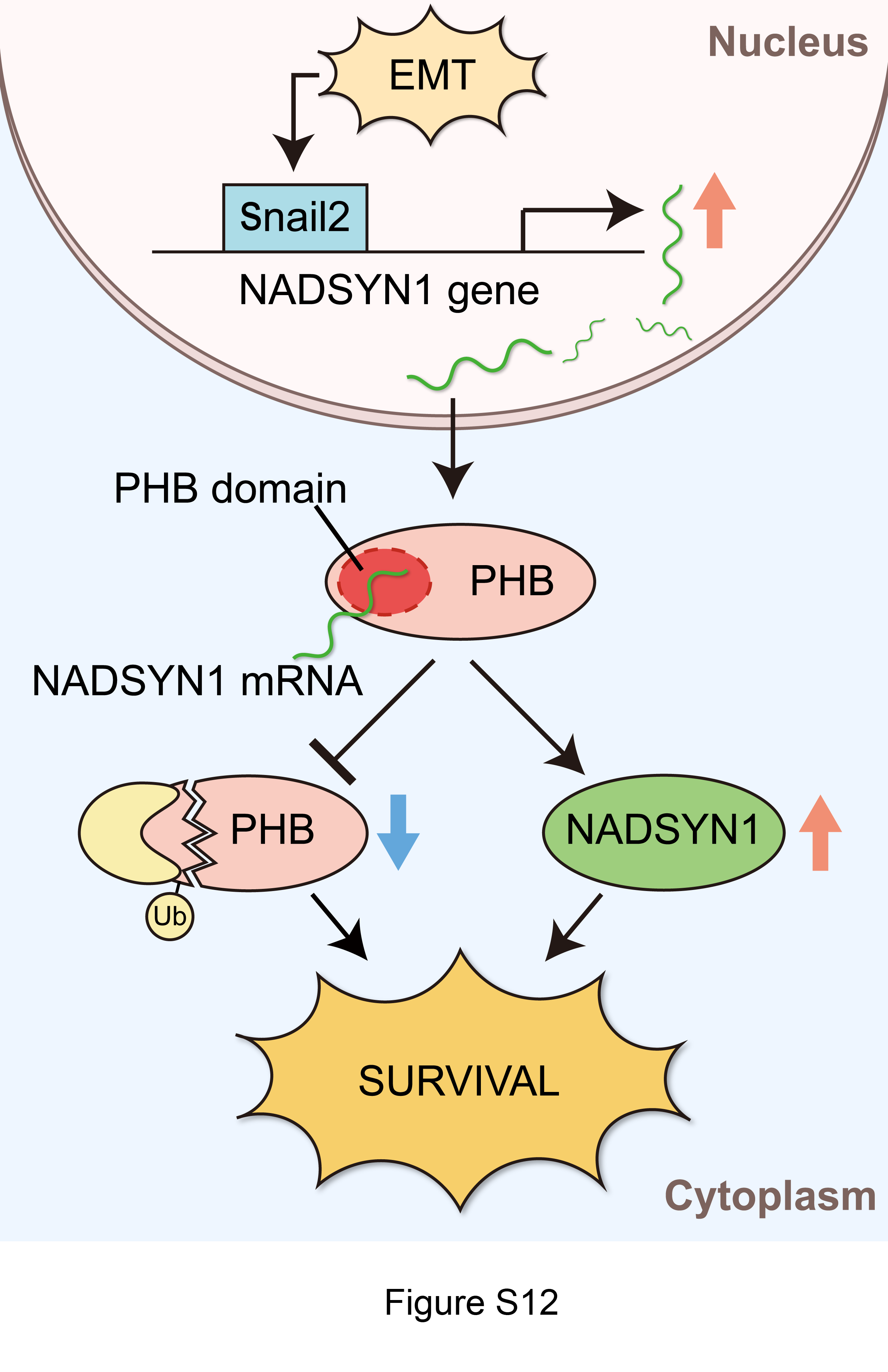
**

**Figure S12. EMT regulated the expression of NADSYN1 *via* Snai2 and Snai2-NADSYN1-PHB axis played an important role in bladder cancer progression.** PHB could directly bind with NADSYN1 mRNA, and PHB domain to be responsible for PHB-NADSYN1 mRNA interaction. Degradation of PHB protein and NADSYN1 mRNA is inhibited by the interaction of PHB-NADSYN1.

**Reference**

1. Tian XP, Wang CY, Jin XH, et al. Acidic Microenvironment Up-Regulates Exosomal miR-21 and miR-10b in Early-Stage Hepatocellular Carcinoma to Promote Cancer Cell Proliferation and Metastasis. *Theranostics*. 2019;9(7):1965-1979. doi:10.7150/thno.30958

2. Xu YZ, Kanagaratham C, Jancik S, Radzioch D. Promoter deletion analysis using a dual-luciferase reporter system. *Methods Mol Biol*. 2013;977:79-93. doi:10.1007/978-1-62703-284-1_7

3. McConkey DJ, Choi W, Marquis L, et al. Role of epithelial-to-mesenchymal transition (EMT) in drug sensitivity and metastasis in bladder cancer. *Cancer Metastasis Rev*. Dec 2009;28(3-4):335-44. doi:10.1007/s10555-009-9194-7

4. Dong P, Jiang L, Liu J, et al. Induction of paclitaxel resistance by ERalpha mediated prohibitin mitochondrial-nuclear shuttling. *PLoS One*. 2013;8(12):e83519. doi:10.1371/journal.pone.0083519

5. Chowdhury I, Thompson WE, Thomas K. Prohibitins role in cellular survival through Ras-Raf-MEK-ERK pathway. *J Cell Physiol*. Aug 2014;229(8):998-1004. doi:10.1002/jcp.24531

6. Ande SR, Xu YXZ, Mishra S. Prohibitin: a potential therapeutic target in tyrosine kinase signaling. *Signal Transduct Target Ther*. 2017;2:17059. doi:10.1038/sigtrans.2017.59

7. Koushyar S, Jiang WG, Dart DA. Unveiling the potential of prohibitin in cancer. *Cancer Lett*. Dec 28 2015;369(2):316-22. doi:10.1016/j.canlet.2015.09.012

8. Swatek KN, Komander D. Ubiquitin modifications. *Cell Res*. Apr 2016;26(4):399-422. doi:10.1038/cr.2016.39

9. Lin J, Zhao L, Zhao S, et al. Disruptive NADSYN1 Variants Implicated in Congenital Vertebral Malformations. *Genes (Basel)*. Oct 14 2021;12(10)doi:10.3390/genes12101615
